# Non-destructive estimation of needle leaf chlorophyll and water contents in Chinese fir seedlings based on hyperspectral reflectance spectra

**DOI:** 10.48130/forres-0024-0021

**Published:** 2024-07-02

**Authors:** Dong Xing, Penghui Sun, Yulin Wang, Mei Jiang, Siyu Miao, Wei Liu, Huahong Huang, Erpei Lin

**Affiliations:** 1 State Key Laboratory of Subtropical Silviculture, Zhejiang A&F University, Hangzhou 311300, Zhejiang, China; 2 Zhejiang International Science and Technology Cooperation Base for Plant Germplasm Resources Conservation and Utilization, Zhejiang A&F University, Hangzhou 311300, China

**Keywords:** *Cunninghamia lanceolata*, hyperspectral imaging, machine learning, needle leaf chlorophyll content, needle leaf water content

## Abstract

Chinese fir is the most important native softwood tree in China and has significant economic and ecological value. Accurate assessment of the growth status is critical for both seedling cultivation and germplasm evaluation of this commercially significant tree. Needle leaf chlorophyll content (LCC) and needle leaf water content (LWC), which are determinants of plant health and photosynthetic efficiency, are important indicators of the growth status in plants. In this study, for the first time, the LCC and LWC of Chinese fir seedlings were estimated based on hyperspectral reflectance spectra and machine learning algorithms. A line-scan hyperspectral imaging system with a spectral range of 870 to 1,720 nm was used to capture hyperspectral images of seedlings with varying LCC and LWC. The spectral data of the canopy area of the seedlings were extracted and preprocessed using the Savitzky-Golay smoothing (SG) algorithm. Subsequently, the Successive Projection Algorithm (SPA) and Competitive Adaptive Reweighted Sampling (CARS) methods were employed to extract the most informative wavelengths. Moreover, SVM, PLSR and ANNs were utilized to construct models that predict LCC and LWC based on effective wavelengths. The results indicated that the CARS-ANNs were the best for predicting LCC, with R²_C_ = 0.932, RSME_C_ = 0.224, and R²_P_ = 0.969, RSME_P_ = 0.157. Similarly, the SPA-ANNs model exhibited the best prediction performance for LWC, with R²_C_ = 0.952, RSME_C_ = 0.049, and R²_P_ = 0.948, RSME_P_ = 0.051. In conclusion, the present study highlights the significant potential of combining hyperspectral imaging (HSI) with machine learning algorithms as a rapid, non-destructive, and highly accurate method for estimating LCC and LWC in Chinese fir.

## Introduction

Chinese fir (*Cunninghamia lanceolata*), the most important native softwood tree mainly distributed in southern China, occupies an important place in the timber industry. It provides essential raw materials for construction, furniture manufacturing and other related industries. The plantation area of Chinese fir covers approximately 11 million hectares, which accounts for around 12.9% of the total plantation forest area in China^[1,2]^. In order to meet the demand for afforestation, more than 500 million seedlings are cultivated every year. It is increasingly important to establish a powerful estimation method to effectively evaluate growth status during seedling cultivation and germplasm phenotyping.

Chlorophyll, the primary pigment in plant photosynthesis, is closely associated with the nutritional status of plants, specifically in terms of its content and spatial distribution. It plays a crucial role in the physiological and developmental health of plants^[3−6]^. Similarly, leaf water content serves as a significant indicator of plant vigor and photosynthetic efficiency, widely used to assess the physiological status of plants^[7,8]^. The correlation between chlorophyll and water content in the needles of Chinese fir seedlings is of paramount importance for the growth of this species. Thus, needles leaf Chlorophyll Content (LCC) and needles leaf Water Content (LWC) can serve as vital indicators for evaluating the growth status of Chinese fir seedlings. However, conventional methods for measuring LCC and LWC in Chinese fir seedlings are destructive, labor-intensive, and rely on chemical reagents in the laboratory. To develop a non-destructive and efficient method for measuring LCC and LWC would be highly valuable for monitoring the growth of seedlings and evaluating the germplasm resources of Chinese fir.

With the development of spectroscopy technology, hyperspectral imaging (HSI) has emerged as a promising tool for measuring traits and evaluating phenotypes in the laboratory, glasshouse, or field^[9,10]^. For instance, researchers have used hyperspectral reflectance data to predict leaf metabolite concentrations and assess drought stress in several agronomic species grown in glasshouses^[11]^. Asaari et al.^[12]^ developed a supervised data-driven method based on the machine learning regression (MLR) algorithm using hyperspectral images, and the best prediction model for four physiological traits was successfully applied in a small-scale phenotyping experiment to study drought stress responses in maize plants. Additionally, many studies have explored the potential of HSI in various aspects of plant phenotyping including estimating physiological and biochemical traits^[13−15]^, detecting plant stress and diseases^[16−18]^ and evaluating plant quality^[19−21]^.

In HSI technology, an appropriate algorithm is critical for establishing the correlation between reflectance spectra and plant traits. Models based on Machine Learning Regression (MLR) are frequently used to predict plant traits from reflectance spectra due to their flexibility and capacity to create responsive input-output relationships^[22,23]^. For example, Xiong et al.,^[24]^ employed Partial Least Squares Discriminant Analysis (PLS-DA), a variable-based regression technique, to construct a predictive model for non-destructive grading and classification of litchi fruits using hyperspectral data ranging from 400 to 1,000 nm. Similarly, Pyo et al.^[25]^ made use of Artificial Neural Network (ANN) and Support Vector Machine (SVM) to effectively classify and quantify cyanobacteria concentrations.

However, to our knowledge, there are no previous reports on the application of HSI on the determination of physiological indicators in Chinese fir. In this study, the objective was to estimate the LCC and LWC in Chinese fir seedlings based on non-destructive HSI and machine learning. To achieve this goal, two estimation models were developed by exploring and validating three MLR algorithms: Partial least squares regression (PLSR), Support Vector Machine (SVM) and Artificial Neural Networks (ANNs). More specific goals were (i) to estimate two targeted physiological traits: LCC and LWC; (ii) to compare and evaluate the superior variable selection method between the Successive Projection Algorithm (SPA) and Competitive Adaptive Reweighted Sampling (CARS) to determine the optimal wavelengths that provide the highest correlation with the two physiological indicators; (iii) to develop robust and accurate estimation models (PLSR, SVM, ANNs) to quantitatively predict the LCC and LWC of Chinese fir seedlings using the optimal wavelengths.

## Materials and methods

### Plant sample preparation

**Figure 1:**
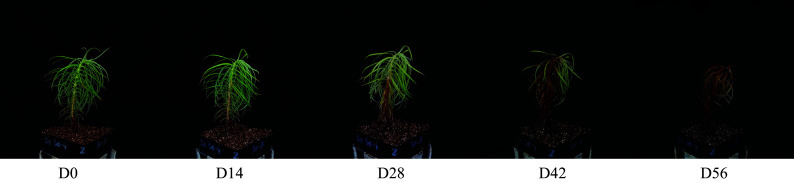
Chinese fir seedlings from different drought treatments.

The seeds were obtained from a bi-clonal seed orchard of Chinese fir clone Long-15 and Min-33 in Kaihua forest farm of Zhejiang Province, China, which were then used to cultivate seedlings in a greenhouse of Zhejiang A&F University. To prepare seedlings with different LWC and LCC, artificial drought stress was used to treat seedlings of about 20 cm in height. One hundred and eighty seedlings were used, and the drought stress was simulated by irrigating with 20% PEG 6000 solution. Each seedling was irrigated with 30 mL of the 20% PEG 6000 solution every 6 d, and the treatment was conducted for 56 d. Hyperspectral data and samples for determining LWC and LCC were collected from 36 seedlings every two weeks in the lab. Accordingly, the seedlings were categorized into five groups: D0 (day 0), D14 (day 14), D28 (day 28), D42 (day 42), and D56 (day 56) (Fig. 1). To enhance the overall robustness of the model, the collected data were divided into two sets: one comprising 126 seedlings for training the regression models, and the other including 54 seedlings for testing the prediction of models.

### Hyperspectral image acquisition

**Figure 2:**
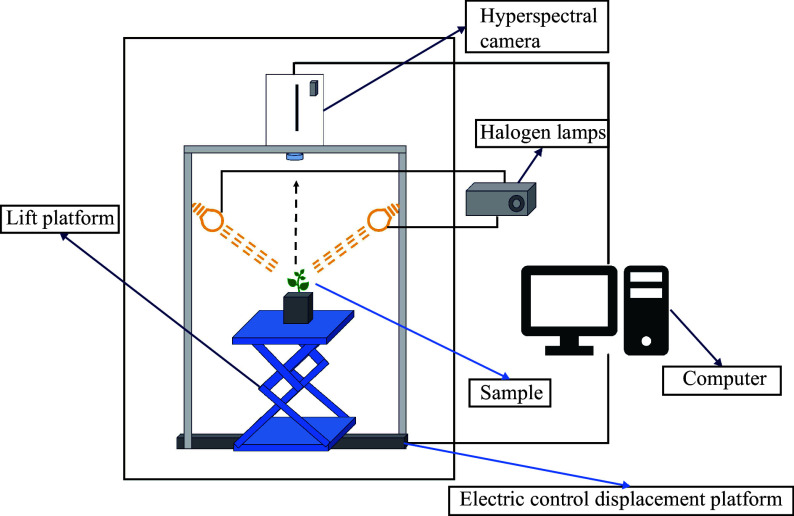
Hyperspectral imaging system.

As shown in Fig. 2, the system for hyperspectral imaging included a NIR hyperspectral imager (GaiaField-N17E, Dualix Spectral Imaging, Sichuan Shuangli Hepu Technology Co., Ltd.), an indoor test chamber (HSIA-BD), a set of four halogen lamps (50 W), a lifting table, a computer, and the supporting software (Optiplex 7080MT/SpecView). The NIR hyperspectral imager had a spectral range spanning from 870 to 1,720 nm, a spatial resolution of 640 pixels, 512 bands, and a spectral resolution of 5 nm. The dimensions of the lifting table were 300 mm × 300 mm, allowing for a lifting range between 90 and 370 mm. To ensure high-quality hyperspectral images of the samples, the conveyor belt was set to move at a speed of 0.6 cm/s with a distance of 25 cm. The sample-to-lens distance was maintained at 30 cm, the angle between the light source and the horizontal plane was set to 60 degrees, and the exposure time was 7 ms.

To avoid the effect caused by uneven light source intensity distribution and dark current during the image collecting process, the white reference image (W) was obtained from white reference panels and the dark reference image (D) was obtained by completely closing the lens of the camera with its opaque cap. The image calibration was performed according to the formula (1):

1 $ R=\dfrac{\mathrm{I}-\mathrm{D}}{\mathrm{W}-\mathrm{D}} $

Where, R represents the corrected image, I represents the original image, W represents the white reference image and D represents the black reference image.

### Destructive measurement of LCC and LWC

After collecting the spectral data, needle leaves were promptly collected to measure the LCC and LWC. To determine the LCC, 0.3 g of canopy needle leaves were chopped into pieces and soaked in 95% ethanol solution in the dark for 24−36 h to extract chlorophyll. The absorbance of the extracted components was then measured using a microplate reader (SpectraMax 190) at wavelengths of 665, 649, and 470 nm^[26]^. The LCC was calculated using the following formula:

2 $ \rm Ca\;(mg/L)=13.95 \times D665-6.88 \times D649 $

3 $ \rm Cb\;(mg/L)=24.96 \times D649-7.32 \times D665 $

4 $ \rm CT\;(mg/L)=Ca+Cb $

5 $ \rm LCC\;(mg/g)=\dfrac{CT \times VT\times BT}{W} $

Where, LCC (mg/g) represents the content of chlorophyll, CT (mg/L) represents the concentrations of chlorophyll a and chlorophyll b, VT (mL) represents the volume of extract solution, BT represents the dilution ratio, and W (g) represents the fresh weight of needle leaves.

For LWC determination, the fresh weight of the needle leaves was initially measured. Subsequently, the needle leaves were subjected to incubation in an oven at 105 °C for 30 min, and then further dried at 80 °C for 48 h until a constant weight was achieved^[27]^. The LWC was calculated using the following formula:

6 $ \rm LWC\;({\%})=\dfrac{M_1-M_2}{M_1}\times 100{\%}$

Where, M_1_ represents fresh needle leaf weight, M_2_ represents drought needle leaf weight.

### Raw spectral data extraction

**Figure 3:**
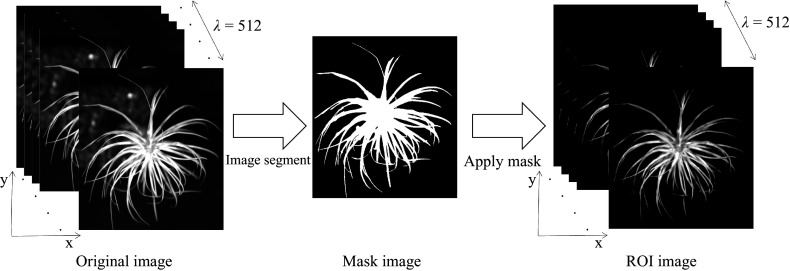
Flow chart of hyperspectral data extraction from NIR hyperspectral images.

Hyperspectral imaging data were analyzed by the ENVI 4.5 software. The canopy area of the seedling was selected as region of interest (ROI) to extract NIR hyperspectral data. A flow chart (Fig. 3) presents the procedure for extracting the NIR hyperspectral data. Initially, a mask was created using a minimum threshold value of 0.45. Subsequently, the original image was masked to yield the target image. Lastly, the raw spectral data were obtained by calculating the mean spectrum of all pixels within the ROI.

### Hyperspectral data preprocessing

The data acquired from the NIR spectrometer contains background information and noise, in addition to sample information. To ensure reliable, accurate, and stable calibration models, it is necessary to preprocess the spectral data before modeling. In the present study, three preprocessing methods were compared and utilized: Savitzky-Golay (SG) smoothing^[28]^, standard normal variate (SNV)^[29]^, and multiplicative scatter correction (MSC)^[30]^. The aim was to select the most optimal approach for preprocessing the spectral data.

### Characteristic wavelength selection

#### Successive Projection Algorithm (SPA)

SPA is a variable-selection method for multivariate calibration, which utilizes projection operations to select a subset of variables with minimum multi-collinearity^[31]^. In the SPA method, multiple linear regression models are created by considering different subsets of the wavelength vector. The wavelengths that result in the lowest Root Mean Square Error (RMSE) are considered the most significant wavelengths^[32]^.

#### Competitive Adaptive Reweighted Sampling (CARS)

CARS, an effective strategy for selecting an optimal combination of key wavelengths present in the full spectrum, is developed based on the principle of 'survival of the fittest' from Darwin's Theory of Evolution^[33]^. Briefly, CARS achieves wavelength selection by establishing PLS models on N (N = 50 in this study) feature subsets generated through the Monte-Carlo (MC) sampling method. Subsequently, the combination of variables with the lowest RMSE during model cross-validation is chosen as the optimal selection^[34,35]^. CARS follows a four-step process in each sampling run: (1) Model sampling using Monte Carlo method; (2) Enforced wavelength selection using an exponentially decreasing function (EDF); (3) Wavelength selection through adaptive reweighted sampling (ARS); (4) Evaluation of the subset through ten-fold cross-validation.

### Machine learning regression

#### Partial Least Squares Regression (PLSR)

PLSR is a widely used methodology in the fields of remote sensing, chemometrics, and spectral data processing. It is particularly useful for handling large datasets that have complex relationships between variables. PLSR is distinguished as a comprehensive full-spectrum approach, leveraging information spanning the entirety of wavelengths within the original spectrum to construct a refined calibration algorithm^[36]^.

#### Support Vector Machine (SVM)

SVM is a widely used and powerful machine learning algorithm that can be applied to both classification and regression tasks^[37]^. Its main principle is to find the optimal hyperplane that can separate data points of different classes in a high-dimensional space^[38]^. This hyperplane is determined through the selection of support vectors, which are the data points closest to the decision boundary. The primary goal of SVM is to maximize the margin, which is the distance between the decision boundary and the nearest data points from both classes. By employing techniques like the kernel trick, SVM can effectively handle nonlinear data by mapping them to a higher-dimensional space to achieve linear separability. This makes SVM a robust and adaptable model that can handle complex datasets.

#### Artificial Neural Network (ANN)

**Figure 4:**
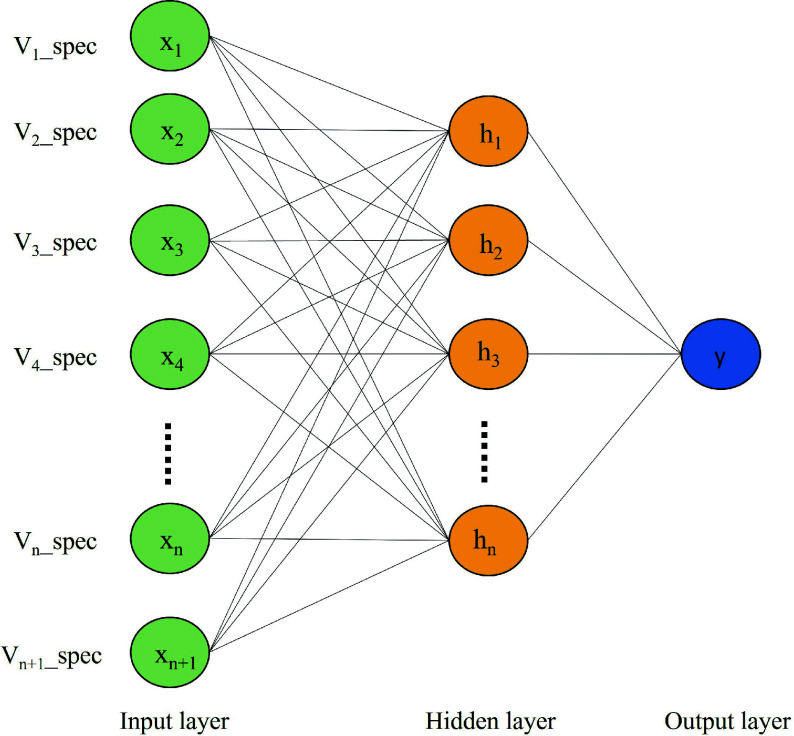
ANNs structure.

Figure 4 demonstrates the structure of the ANN model, comprising three essential layers: input, hidden, and output layers. The input layer plays a crucial role in seamlessly integrating with external systems, assimilating external data for a harmonious connection. On the other hand, the output layer disperses the predictive results of the model into the external environment, with its neuron count being intricately linked to the specific task under consideration. In contrast, the often disregarded hidden layer acts as a mediator, bridging the gap between the input and output layers. Neurons within this layer incorporate activation functions, to introduce nonlinear dynamics during the transmission of information. This intermediary layer assumes pivotal responsibility within the overall model, enabling sophisticated abstraction and subtle feature extraction through progressive refinement and transformation of input data. The fundamental principle of hierarchical transmission and processing empowers neural networks to meticulously capture inherent data correlations, resulting in improved accuracy in predictive and analytical results^[39]^.

### Model evaluation

By employing r-squared of the calibration set (R^2^_C_), r-squared of the prediction set (R^2^_P_), root mean square error of the calibration set (RMSE_C_) and root mean square error of the prediction set (RMSE_P_), the evaluation of the model's predictive capability was conducted. Through a thorough examination of both the modeling and validation accuracies, the most optimal prediction model can be determined. The calculation formulas for R^2^ and RMSE can be defined as follows:

7 $ {R}^{2}=1-\dfrac{{\sum _{i}^{n}\left(yi-\hat{y}i\right)}^{2}}{{\sum _{i}^{n}\left(yi-\overline{y}i\right)}^{2}} $

8 $ RMS E=\sqrt{\dfrac{{\sum _{i=1}^{n}\left(yi-\hat{y}i\right)}^{2}}{n}} $

Where, $ yi $ and $ \hat{y}i $ are the measured and predicted values, respectively. $ \overline{y}i $ is the average of the measured value, n is the total number of sample test data sets.

## Results and discussion

### Measurement of LCC and LWC

**Figure 5:**
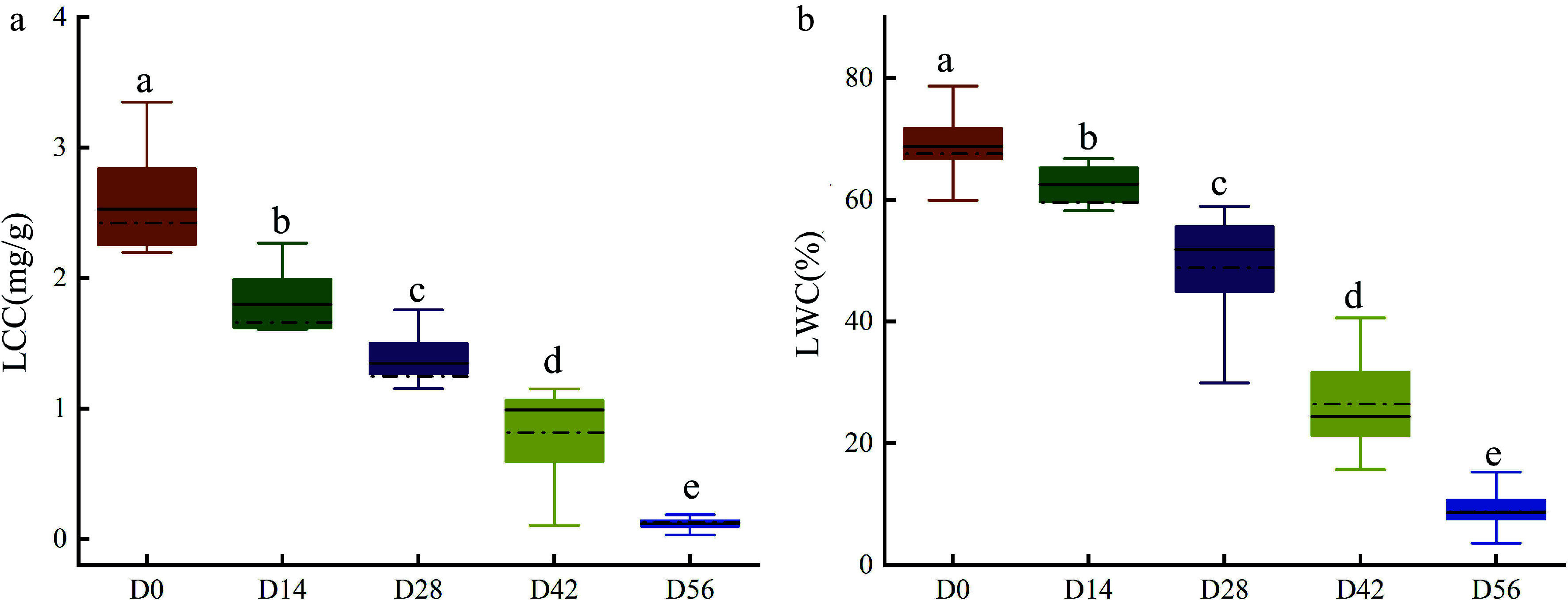
Measured (a) LCC and (b) LWC in Chinese fir seedlings of the five drought treatment groups.

After capturing the hyperspectral images, the LCC and LWC of Chinese fir seedlings were destructively determined immediately. As shown in Fig. 5, the LCC and LWC gradually decreased with the extension of drought time, and significant differences were observed between different drought treatments. The average LCC of seedlings in D0 was 2.4 mg/g, while in D56 it was only 0.1 mg/g (Fig. 5a). Similarly, the average LWC of seedlings in D0 was 68%, whereas in D56 it was only 9% (Fig. 5b). These measured data were used as the ground truth for model training and validation.

### Spectral features

**Figure 6:**
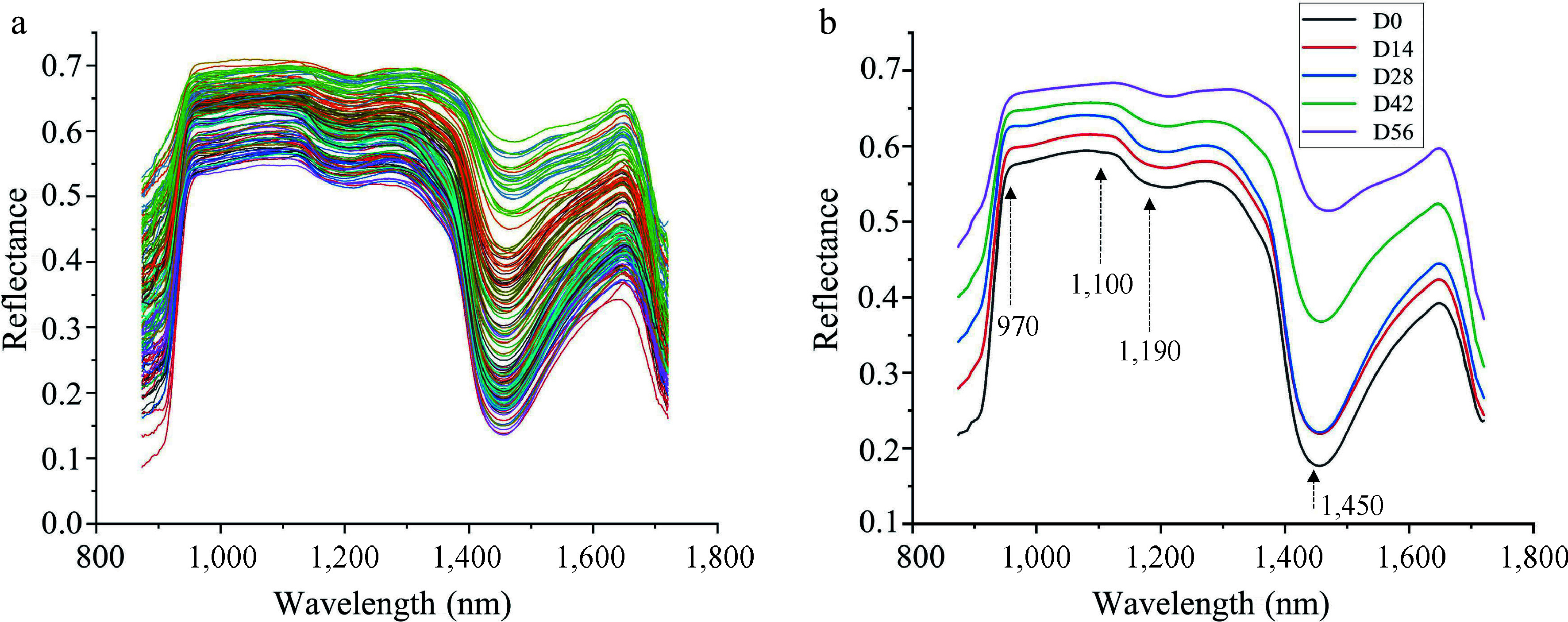
(a) Raw reflectance curves and (b) average reflectance curves of Chinese fir seedlings with different LCC and LWC.

The raw and average reflectance spectral curves for seedlings with different LCC and LWC were shown in Fig. 6a & b, respectively. As can be seen in the figure, the spectral curves of all seedlings showed a similar pattern (Fig. 6a), while the spectral data was sensitive to the changes in LCC and LWC, resulting in fluctuating reflectance with the changes of LCC and LWC (Fig. 6b). The absorption peaks around 1,450 and 970 nm were observed in all datasets (Fig. 6b), which are related to the O–H first and second overtones of water, respectively^[40−43]^. Similarly, an absorption peak near 1,100 nm appeared in all samples is associated with the second overtone of N-H in chlorophyll^[44]^. Additionally, a broad absorption peak near 1,190 nm caused by the C-H stretching vibration of CH_3_^[5]^. The variation in this spectral reflectance could potentially help discriminate the physiochemical properties between samples^[45]^.

### Hyperspectral data preprocessing

**Figure 7:**
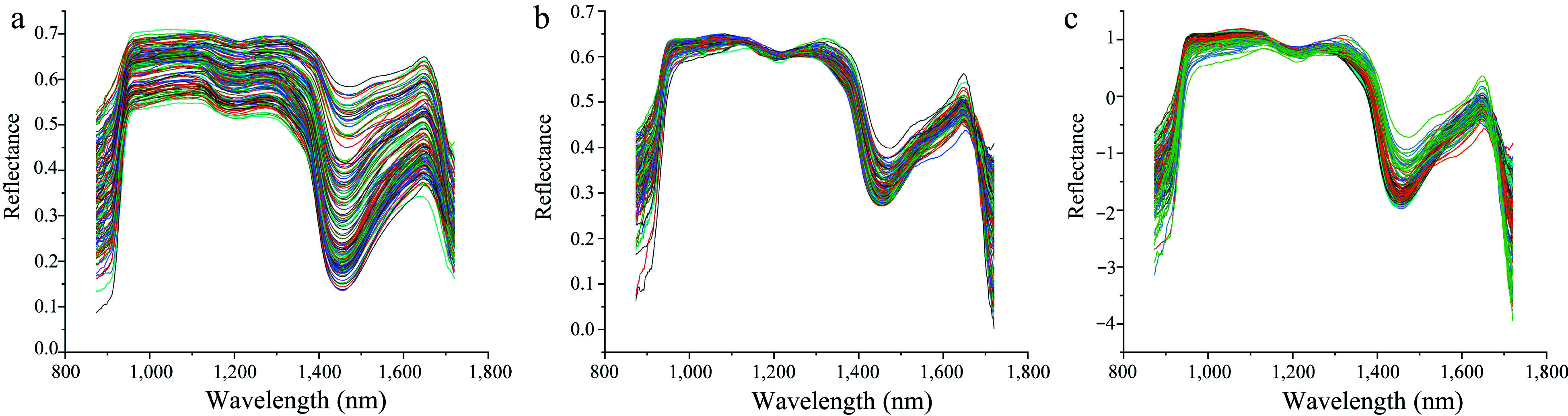
Comparison of different preprocessing methods for hyperspectral data. (a) Hyperspectral data preprocessed by SG. (b) Hyperspectral data preprocessed by MSC. (c) Hyperspectral data preprocessed by SNV.

It is necessary to perform spectral preprocessing to remove noise and invalid information introduced by environmental factors and instrument noise^[46]^. Many spectral preprocessing methods have been reported, and the choice of preprocessing method depends on the nature of the spectrum and the component features that need to be predicted^[47]^. In the present study, the raw hyperspectral data were pre-processed using SG, MSC and SNV, respectively. As shown in Fig. 7, SG could effectively eliminate spectral deviation caused by different scattering levels and retain the spectral characteristics (Fig. 7a), while the MSC and SNV changed the spectral curves by removing many spectral information (Fig. 7b & c). Further evaluation on these pre-processed data was also performed by employing the partial least squares discriminant analysis (PLS-DA) to develop the multivariate models. As shown in Table 1, the SG derivative exhibited the best results for predicting LCC with an R^2^_C_ of 0.9166, RMSE_C_ of 0.2587, R^2^_P_ of 0.8616, and RMSE_P_ of 0.3547. Furthermore, the SG derivative-PLS-DA model also achieved the best results for LWC prediction with an R^2^_C_ of 0.9350, RMSE_C_ of 0.0552, R^2^_P_ of 0.9048, and RMSE_P_ of 0.0661. These results indicated that SG pre-processing could enhance the correlation between spectrum and measured data. Based on these findings, the SG pre-processed spectral data were chosen as the optimal datasets for subsequent prediction analysis.

**Table 1:** Influence of different preprocessing methods on LCC and LWC prediction.

Index	Preprocessing	Calibration set			Prediction set	
		R^2^_C_	RMSE_C_		R^2^_P_	RMSE_P_
LCC	None	0.8943	0.2835		0. 8198	0.3268
	MSC	0.8140	0.3654		0.7756	0.4405
	SG	0.9166	0.2587		0.8616	0.3547
	SNV	0.8322	0.3491		0.7135	0.5053
LWC	None	0.9023	0.0540		0.8904	0.0771
	MSC	0.8983	0.0694		0.7832	0.1027
	SG	0.9350>	0.0552		0.9048	0.0661
	SNV	0.9120	0.0459		0.8714	0.1073

### Selection of characteristic wavelengths

**Figure 8:**
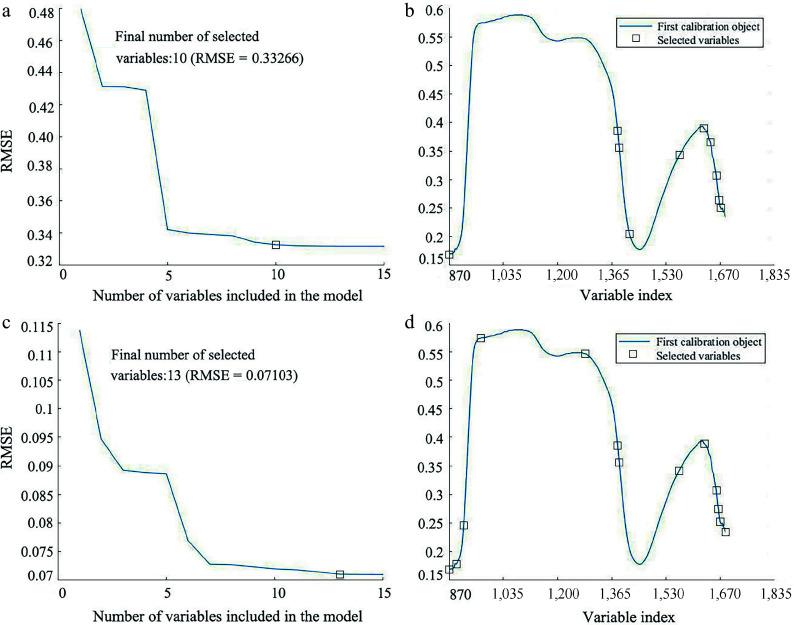
Result of applying SPA wavelength selection on the SG pre-processed spectrum for predicting LCC and LWC. (a) Variation of RMSE vs the number of wavelengths, and (b) the selected wavelengths for LCC prediction. (c) Variation of RMSE vs the number of wavelengths, and (d) the selected wavelengths for LWC prediction.

To improve the prediction performance of the model and reduce redundancy and collinearity in the spectral data, the SPA and CARS selection algorithms were utilized to extract characteristic wavelengths from the SG preprocessed spectra. Figure 8 illustrates the results of the SPA algorithm for wavelength selection in LCC and LWC prediction. The variation of RMSE relative to the number of wavelengths is depicted in Fig. 8a & c. It can be observed that the RMSE decreased as the number of included variables increased. This decreasing trend continued until the number of included wavelengths reached 10 (RMSE = 0.33266) for LCC prediction and 13 (RMSE = 0.07103) for LWC prediction, respectively. Therefore, 10 and 13 characteristic wavelengths were selected for LCC and LWC prediction, respectively. Figure 8b & d shows the distribution of the selected characteristic wavelengths.

**Figure 9:**
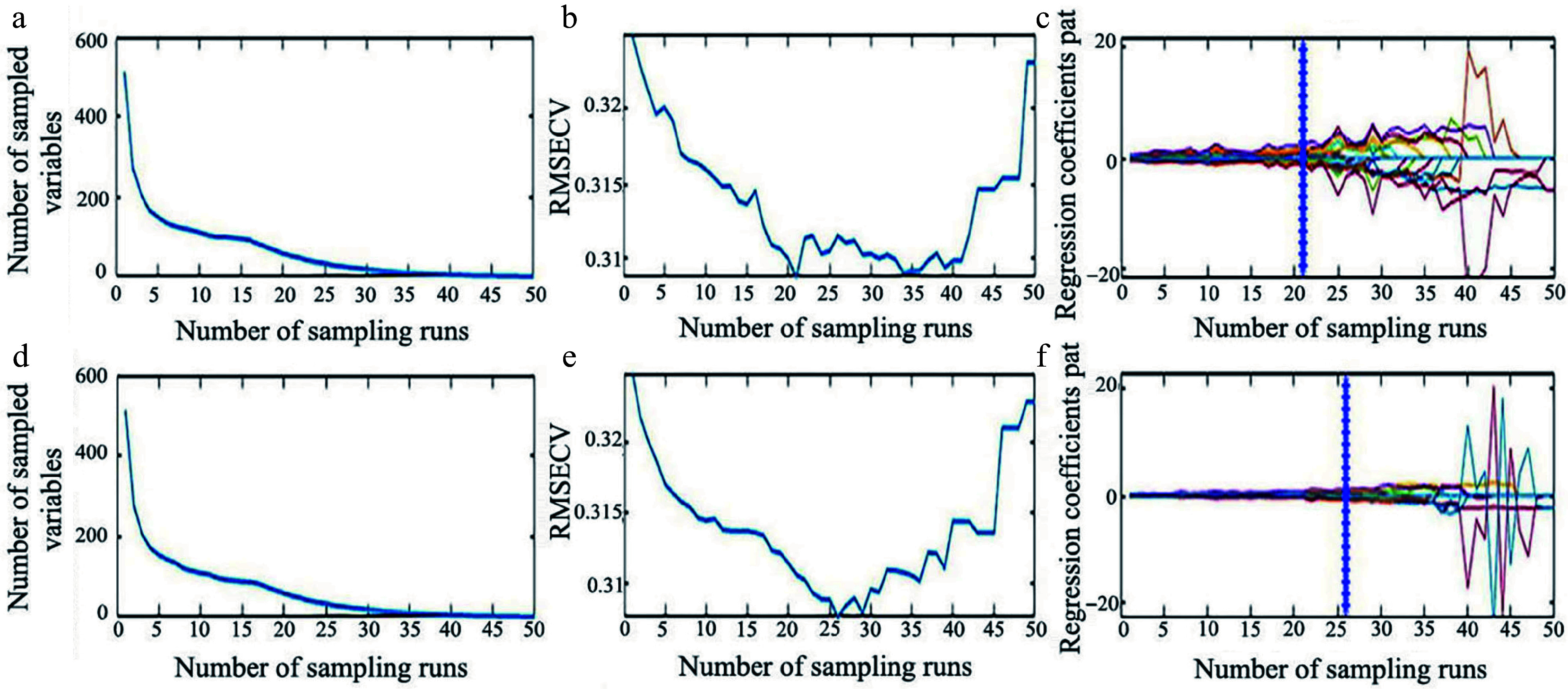
Process of extracting characteristic wavelength by CARS. (a) Number of preferred characteristic wavelength variables, (b) the root mean square error of cross-validation variation, and (c) regression coefficient path map for LCC. (d) Number of preferred characteristic wavelength variables, (e) the root mean square error of cross-validation variation, and (f) regression coefficient path map for LWC.

The CARS algorithm was also reported as an effective wavelength selection method in various studies^[48−50]^. The processes of applying the CARS algorithm on the SG preprocessed data for LCC and LWC prediction are presented in Fig. 9. As shown in Fig. 9a, it can be seen that the number of sampled wavelengths decreased rapidly during the initial step of MC sampling. However, the decreasing trend became milder after the first sharp fall during the refined selection, which can be attributed to the exponentially decreasing function (EDF) in feature selection. The variations of the RMSE value of tenfold cross-validation are shown in Fig. 9b. The RMSE value decreased quickly until the sampling run of 21, after which it increased again. The optimal number of wavelengths, indicated by the vertical star line in Fig. 9c, was 53 out of the 512 wavelengths (approximately 10.35%). A similar process was followed for the prediction of LWC using CARS (Fig. 9d−f). As shown in Fig. 9f, at the 26^th^ sampling run, 29 characteristic wavelengths (approximately 5.66% of 512 bands) were obtained.

The wavelengths selected by SPA and CARS algorithms for the prediction of LCC and LWC in this study are listed in Table 2. For LCC, the characteristic wavelengths are mostly concentrated in the band range of 870−960, 1,100−1,200, and 1,400−1,700 nm (Table 2). Among the selected wavelengths, the wavelengths distributed between 1,425−1,440 and 1,600−1,700 nm are similar to the characteristic wavelengths of 1,420 and 1,694 nm for LCC in Toona sinensis samples^[26]^. Additionally, the selected wavelengths from 1,100−1,200 nm have shown associations with the vibrations of the C-H and N-H groups found in chlorophyll^[51]^. As for the LWC, the chosen wavelengths of 873.5, 881.9, 885.3, 895.3, 1,289.9, 1,389.3, 1,440.5, 1,549.4, 1,575.7, 1,580.7, 1,676.1, 1,689.2, and 1,702.3 nm display similarities to the characteristic wavelengths of 871.61, 880.42, 893.5, 1,285.05, 1,395.19, 1,587.44, 1,662.2, and 1,703.41 nm for water content in tea needle leaves^[52]^. The chosen wavelength of 968.8 nm is associated with the O-H stretching overtones, including the first, second, and third overtones^[53]^. The chosen wavelengths of 1,213.6, 1,394.2, and 1,653, 1,664.6 nm show similarity to the wavelengths of 1,213.69, 1,395.72, 1,659.36, and 1,662.5 nm reported by Song et al. for LWC in rice samples^[54]^.

**Table 2:** Characteristic wavelengths selected by SPA and CARS.

Selection method	Index	Number of feature bands	Selected wavelengths (nm)
SPA	LCC	10	873.5, 1,387.6, 1,394.2, 1,425.7, 1,577.4, 1,651.4, 1,671.1, 1,689.2, 1,697.4, 1,702.3
	LWC	13	873.5, 895.3, 917, 968.8, 1,289.9, 1,389.3, 1,394.2, 1,575.7, 1,653, 1,689.2, 1,695.8, 1,700.7, 1,702.3
CARS	LCC	53	880.2, 881.9, 883.6, 885.3, 890.3, 953.8, 955.5, 957.1, 958.8, 962.2, 967.2, 1,137.2, 1,138.8, 1,142.2, 1,152.2, 1,153.8, 1,158.8, 1,162.1, 1,213.6, 1,225.3, 1,231.9, 1,233.6, 1,424, 1,430.6, 1,432.3, 1,433.9, 1,435.6, 1,542.8, 1,544.4, 1,546.1, 1,547.7, 1,549.4, 1,552.7, 1,557.6, 1,559.3, 1,560.9, 1,565.9, 1,567.5, 1,574.1, 1,580.7, 1,662.9, 1,664.6, 1,666.2, 1,669.5, 1,671.1, 1,672.8, 1,674.4, 1,676.1, 1,677.7, 1,684.3, 1,699, 1,700.7, 1,702.3
	LWC	29	881.9, 883.6, 885.3, 958.8, 1,213.6, 1,231.9, 1,233.6, 1,427.3, 1,433.9, 1,435.6, 1,440.5, 1,549.4, 1,552.7, 1,554.3, 1,556, 1,557.6, 1,560.9, 1,562.6, 1,565.9, 1,567.5, 1,580.7, 1,664.6, 1,669.5, 1,671.1, 1,672.8, 1,674.4, 1,676.1, 1,700.7, 1,702.3

### Modeling based on full wavelengths and selected wavelengths

Previous studies have shown the great potential of machine learning models in predicting chlorophyll content and water content of different plant samples^[55,56]^. In this study, full wavelengths and characteristic wavelengths selected by SPA and CARS were utilized to establish prediction models, respectively. The prediction models were thus built using three machine learning algorithms: PLSR, SVM, and ANNs. The regression results of the established models were evaluated based on the determination coefficient (R^2^) and RMSE. As shown in Table 3, the models based on wavelengths selected by SPA and CARS exhibited better performance compared to those based on full-band spectral data. This indicates that SPA and CARS can reduce the redundancy of input variables in the model and help improve its accuracy.

**Table 3:** The prediction results of LCC and LWC by PLSR, SVM and ANNs models full and selected wavelengths.

Index	Model	Number of bands	Calibration set			Prediction set	
			R^2^_C_	RMSE_C_		R^2^_P_	RMSE_P_
LCC	Full-PLSR	512	0.797	0.363		0.839	0.359
	SPA-PLSR	10	0.804	0.360		0.842	0.354
	CARS -PLSR	53	0.805	0.358		0.843	0.353
	Full-SVM	512	0.830	0.350		0.820	0.392
	SPA-SVM	10	0.812	0.380		0.770	0.450
	CARS-SVM	53	0.830	0.360		0.820	0.397
	Full-ANNs	512	0.930	0.240		0.870	0.349
	SPA-ANNs	10	0.920	0.267		0.924	0.300
	CARS-ANNs	53	0.932	0.224		0.969	0.157
LWC	Full-PLSR	512	0.856	0.070		0.901	0.082
	SPA-PLSR	13	0.804	0.360		0.842	0.354
	CARS -PLSR	29	0.858	0.072		0.901	0.079
	Full-SVM	512	0.873	0.079		0.930	0.060
	SPA-SVM	13	0.850	0.090		0.920	0.062
	CARS-SVM	29	0.858	0.078		0.929	0.063
	Full-ANNs	512	0.954	0.187		0.873	0.348
	SPA-ANNs	13	0.952	0.049		0.948	0.051
	CARS-ANNs	29	0.952	0.050		0.940	0.058

**Figure 10:**
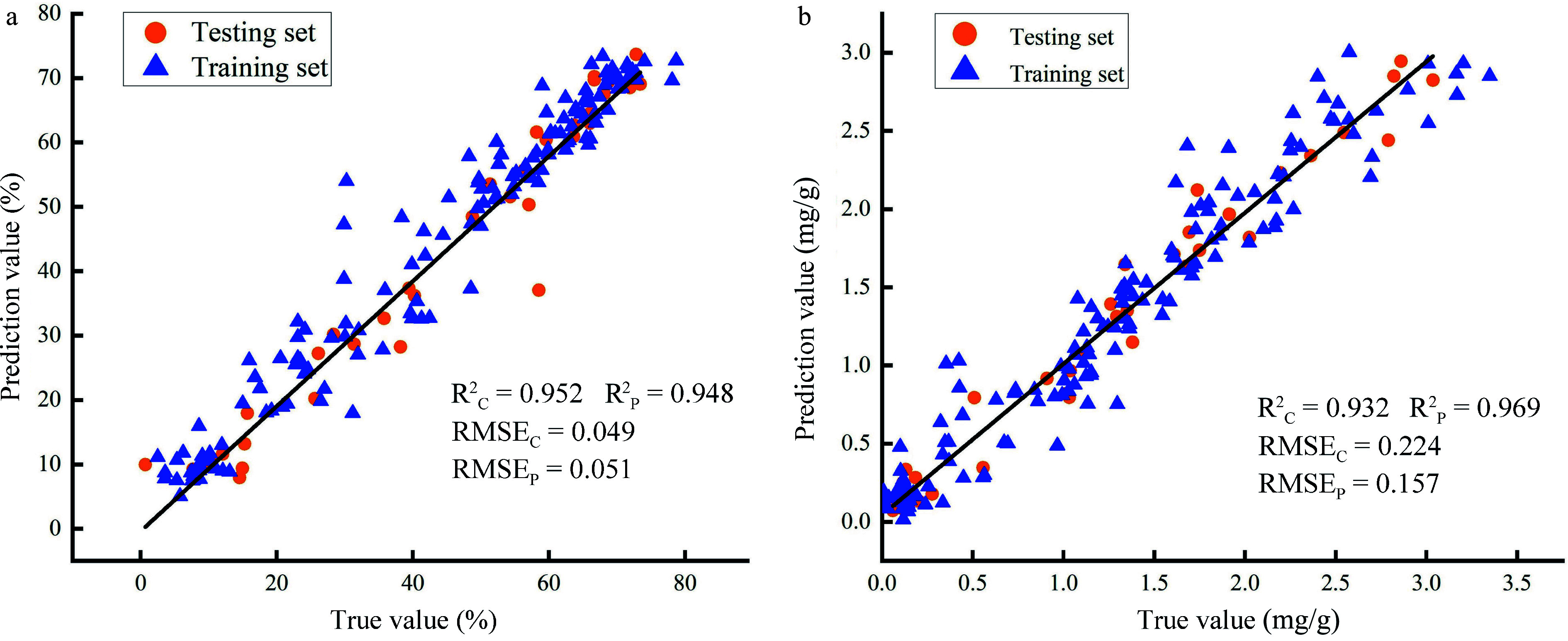
Correlation analysis between true and prediction values. (a) Prediction accuracy of SPA-ANNs model for LWC; (b) Prediction accuracy of CARS-ANNs model for LCC.

For LCC prediction, it is obvious that the CARS-ANNs model, using 53 characteristic wavelengths as input has achieved the best performance with the values of R²_C_ and R²_P_ reaching 0.932 and 0.969, and RMSE_C_ and RMSE_P_ are 0.224 and 0.157, respectively (Table 3). Meanwhile, the SPA-ANNs model, requiring 13 feature wavelengths exhibited the most accurate prediction for LWC, and the obtained R²_C_, RMSE_C_, and R²_P_ and RMSE_P_ were 0.952, 0.049, and 0.948, 0.051, respectively (Table 3). Furthermore, the performances of the CARS-ANNs and SPA-ANNs models were verified by correlation analysis. The result also showed high prediction accuracy of both models for LCC and LWC, respectively (Fig. 10). In summary, the use of wavelength selection techniques significantly enhanced the prediction accuracy of LWC and LCC. On the other hand, among all the established models, ANN-based models achieved better performance than SVM and PLSR (Table 3). This suggests the great potential of ANN in the phenotypic evaluation of plants^[44,57]^.

## Conclusions

This study aimed to investigate the application of hyperspectral imaging in predicting the needle leaf chlorophyll content (LCC) and needle leaf water content (LWC) of Chinese fir seedlings. Reflectance images were captured from seedlings with varying levels of LCC and LWC. Various spectral data preprocessing algorithms were applied, followed by two wavelength selection methods, to prepare the necessary variables for establishing prediction models. The results showed that the Savitzky-Golay (SG) preprocessing method was the most effective at removing background noise and interference factors. Additionally, the wavelengths selected by the Successive Projections Algorithm (SPA) were identified to be the optimal features for predicting LWC, while the wavelengths selected by the Competitive Adaptive Reweighted Sampling (CARS) method were the most suitable variables for predicting LCC. Eventually, the CARS-ANNs model achieved the highest performance in predicting LCC, with an R^2^_P_ value of 0.941 and RMSE_P_ value of 0.240. On the other hand, the SPA-ANNs model showed the best performance in predicting LWC, with an R^2^_P_ value of 0.952 and RMSE_P_ value of 0.049. These results suggest that combining hyperspectral imaging with machine learning models enables the fast, non-destructive, and highly accurate detection of LCC and LWC in Chinese fir seedlings. This study introduces a new method for rapidly and non-destructively evaluating physiological traits for the phenotyping, breeding, and cultivation of conifers like Chinese fir.

## Author contributions

The authors confirm contribution to the paper as follows: study conception: Xing D, Sun P, Lin E; data collection: Xing D, Jiang M, Sun P; data curation: Wang Y, Miao S, Liu W; writing code, conducting and designing experiments: Xing D, Sun P; writing original manuscript: Sun P, Huang H, Lin E. All authors reviewed the results and approved the final version of the manuscript.

## Data availability

The datasets generated during and/or analyzed during the current study are available from the corresponding author on reasonable request.
